# Supplementary material for: Long-Term High-Fat Diet Consumption Induces Cognitive Decline Accompanied by Tau Hyper-Phosphorylation and Microglial Activation in Aging
Source: Nutrients. 2023 Jan 3;15(1):250. doi: 10.3390/nu15010250 (PMC9823602; doi:10.3390/nu15010250)
Supplement: Supplementary file 1 [file nutrients-15-00250-s001.zip › nutrients-2079407-supplementary.pdf]

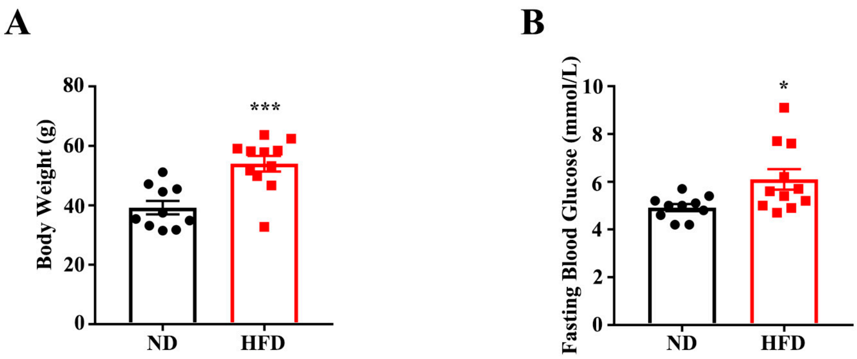

Figure S1. Long-term HFD feeding increases body weight and fasting blood glucose of aged mice. Body weight (A) and fasting blood glucose (B) of mice after 10-month ND or HFD feeding. Data are presented as Mean ± SEM, (n=10-11) for each group. * $p < 0.05$; ***$p < 0.001$ vs. ND group.

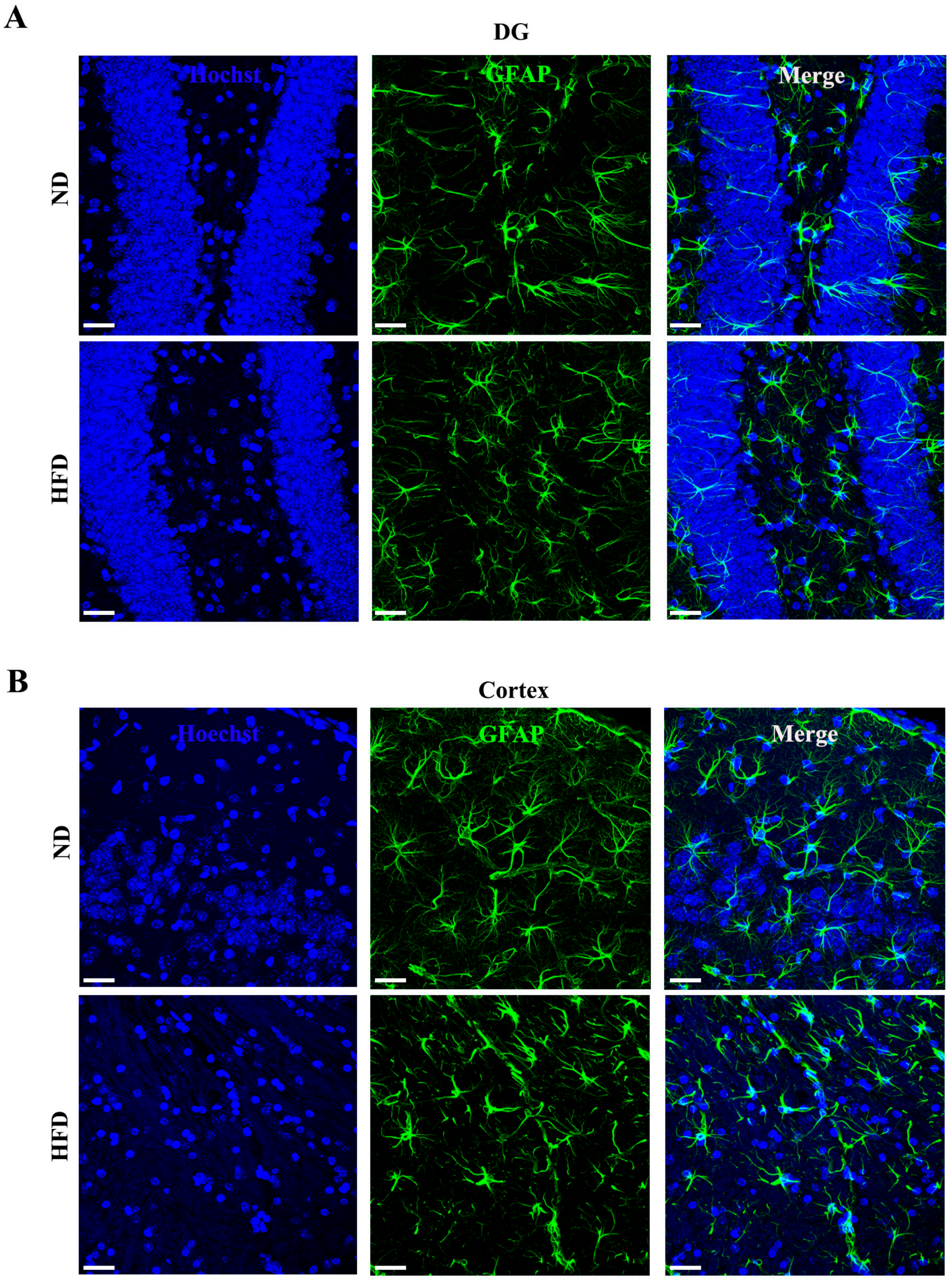

Figure S2. Long-term HFD feeding does not activated astrogliosis in aged mice. (A and B) The representative images of astrocyte in DG and Cortex area. (scale bar = 30 μm).
